# PD98059 Protects Brain against Cells Death Resulting from ROS/ERK Activation in a Cardiac Arrest Rat Model

**DOI:** 10.1155/2016/3723762

**Published:** 2016-03-16

**Authors:** Phuong Anh Nguyen Thi, Meng-Hua Chen, Nuo Li, Xiao-Jun Zhuo, Lu Xie

**Affiliations:** ^1^Department of Physiology, Pre-Clinical Science, Guangxi Medical University, Nanning, Guangxi 530021, China; ^2^Institute of Cardiovascular Diseases, The First Affiliated Hospital of Guangxi Medical University, Nanning, Guangxi 530021, China

## Abstract

The clinical and experimental postcardiac arrest treatment has not reached therapeutic success. The present study investigated the effect of PD98059 (PD) in rats subjected to cardiac arrest (CA)/cardiopulmonary resuscitation (CPR). Experimental rats were divided randomly into 3 groups: sham, CA, and PD. The rats except for sham group were subjected to CA for 5 min followed by CPR operation. Once spontaneous circulation was restored, saline and PD were injected in CA and PD groups, respectively. The survival rates and neurologic deficit scores (NDS) were observed, and the following indices of brain tissue were evaluated: ROS, MDA, SOD, p-ERK1/2/ERK1/2, caspase-3, Bax, Bcl-2, TUNEL positive cells, and double fluorescent staining of p-ERK/TUNEL. Our results indicated that PD treatment significantly reduced apoptotic neurons and improved the survival rates and NDS. Moreover, PD markedly downregulated the ROS, MDA, p-ERK, and caspase-3, Bax and upregulated SOD and Bcl-2 levels. Double staining p-ERK/TUNEL in choroid plexus and cortex showed that cell death is dependent on ERK activation. The findings in present study demonstrated that PD provides neuroprotection via antioxidant activity and antiapoptosis in rats subjected to CA/CPR.

## 1. Introduction

Cardiac arrest (CA) remains a leading cause of death and long-term disability worldwide, thus representing a major concern to public health and the economy [1]. Despite many years of laboratory and clinical research, treatment of postcardiac arrest has not reached therapeutic success. Survival rate and neurological injury outcome following CA and cardiopulmonary resuscitation (CPR) remain poor [2]. The patients with successful return of spontaneous circulation (ROSC) have neurological deficits, 80% are comatose or with persistent vegetative state, with only 3% to 7% able to return to their previous level of brain function [3, 4]. The pathogenesis of cerebral ischemia/reperfusion (I/R) injury is very complex and remains incompletely understood; overproduction of reactive oxygen species (ROS) plays vital role in I/R brain damage [5–7]. Overproduction of ROS can cause oxidative damage to biomolecules (lipid, protein, and DNA) in membrane and nucleus, eventually leading to cell death [8]. Furthermore, ROS is able to activate kinases or inhibit phosphatases resulting in stimulation of signaling pathways [9, 10]. ERK belongs to a family of mitogen-activated protein kinases (MAPKs) which is one example for ROS-regulated kinases [11, 12]. ROS can inhibit protein phosphatases resulting in the activation of the ERK1/2 signaling pathway [12]. ROS are also able to stimulate directly growth receptors such as EGFR and PDGFR, which induces the activation of both Ras and ERK1/2 pathways [13]. Evidences showed that ERK1/2 pathway is phosphorylated in the damaged brain after ischemia in animal model [14, 15]. Inhibitors of MEK/ERK 1/2, PD98059 (PD), and U0126 reduce infarct volume and cell death in transient occlusion of the middle cerebral artery in mice [14–16]. By contrast, some research results indicated that the inhibition of ERK1/2 activity aggravates neuronal injury and accelerates apoptosis [17, 18]. These differences in outcome resulting from MEK1/2 inhibition depend on various reasons, including the nature and severity of injury, the drug dosing, the observing time point, and the cell type expressing activated ERK1/2. So, further research is required to define the roles of ERK pathway involved in cerebral I/R pathophysiological process with various kinds of models.

CA rat models that closely mimic patient cardiac arrest circumstances may be useful in studying the mechanisms of cerebral ischemia injury as well as the efficacy of neuroprotective drugs [19, 20]. Thus, in the present study we used a rat CA/CPR model to assess antioxidant and antiapoptosis effects of PD treatment by detecting the survival rates, NDS, ROS, MDA, and SOD level and the expression of p-ERK1/2 and ERK as well as the apoptosis-associated proteins caspase-3, Bax, and Bcl-2.

## 2. Experimental Section

### 2.1. Animal Preparation

Adult Sprague-Dawley male rats (300–400 g) were obtained from the Experimental Animal Center of Guangxi Medical University (China, Nanning). All animal experiments were performed in accordance with the Guidelines for the Care and Use of Laboratory Animals. This study protocol had been examined and approved by the animal ethics committee of Guangxi Medical University.

### 2.2. Experimental Cardiac Arrest Rat Model

The rat cardiac arrest model was established according to a method reported by Chen et al. [21]. All rats, which were fasted for 12 hours but had free access to water before operation, were injected with pentobarbital sodium (45 *μ*g/g, i.p.) and supplemented by additional doses of 10 *μ*g/g at an hour interval. After endotracheal intubation, cardiac rhythm was monitored with a standard lead II ECG. Two 20-gauge catheters, filled with saline containing 5 IU/mL of sodium heparin, were, respectively, inserted into the left femoral artery for haemodynamic monitoring and the left femoral vein for drug delivery. Pressure transducers were connected to a four-channel physiological recorder (BL-420 E Bio-Systems, Chengdu Technology & Market Co. Ltd., China). After 5-minute baseline EEG and physiologic measurements, the rats were induced CA by alternating current AC (12 V) from a stimulator (Chengdu Technology & Market Co. Ltd., China) through a pacing electrode put in esophagus. CA was defined as a loss of aortic pulsation or aortic pulse pressure <10 mmHg. Five minutes after CA, CPR was initiated with effective ventilation (TV 8 mL/kg, respiration rate 40/min, and PEEP 0 cm H_2_O) using a volume-controlled small animal ventilator (DH-150, The Medical Instrument Department of Zhejiang University, China), oxygenation (100% O_2_), epinephrine (0.02 mg/kg, i.v.), and sternal chest compression (180 compressions/min). Restoration of spontaneous circulation (ROSC) was defined as an unassisted pulse with a mean arterial pressure (MAP) of ≥50 mmHg for ≥1 min. Mechanical ventilation was withdrawn, when spontaneous breathing occurred at ≥40 breaths per minute for ≥1 min within one hour after ROSC and the blood pressure was stable or increased gradually. The rats were injected intravenously with saline (CA group) or ERK inhibitor PD 0.3 mg/kg in DMSO solution (PD group) immediately after ROSC. After resuscitation, rats were placed alone in a cage with dry bedding and housed in an air-conditioned and peaceful room (room temperature 27°C). Rats had free access to water and food. The rats were divided into two experiments after ROSC, one experiment to examine the survival rates and NDS, and the other one for brain harvest to do biochemical analysis.

### 2.3. Survival Observation and Neurological Evaluation

Thirty-two rats (*n* = 13 for CA and PD groups, resp., *n* = 6 for sham group) were used for the survival observation and neurological evaluation. The survival rate and neurologic deficit evaluation at 12, 24, 48, and 72 h were measured during survival observation without using pain relievers, anesthesia, and euthanasia after ROSC. The neurologic deficit scores (NDS) measures level of arousal, cranial nerve reflexes, motor function, and simple behavioral responses and has a range of 0–80 (Table 2). This experiment was defined as the 72 h NDS score. We prespecified the NDS cut-off for good (NDS ≥ 60) and poor (NDS < 60) outcome which represents a level of neurologic function required for independent function [22].

### 2.4. Tissue Preparation

Fifty-four rats (*n* = 24 for CA and PD groups, resp., *n* = 6 for sham group) were used for harvesting tissues preparation. Animals were sacrificed according to the time points of different groups. Therefore, animals in the sham group were sacrificed at 72 h after the sham operation. The rats were euthanized with intravenous injection of 2 mL of saturated potassium chloride solution and brains were immediately excised. The left brains were fixed in 4% buffered paraformaldehyde for immunostaining and terminal deoxynucleotidyl transferase-mediated biotinylated UTP nick end labeling assay (TUNEL). The right brains were used for ROS detection, oxidative stress detection, and western blot.

### 2.5. ROS Detection

Dihydroethidium (DHE) has been commonly used to detect cytosolic superoxide. DHE can bind irreversibly to the double-stranded DNA, causing amplification of a red fluorescent signal, and appears as punctate nuclear staining indicating ROS production [23]. Harvested brain samples were immediately frozen at −20°C and cut by CM1950 Cryostat Microtome (made in German Leica). Serial, coronal frozen sections (5 *μ*m thick) were fixed on slices before being incubated with DHE (10 *μ*mol/L) in PBS in a light-protected, humidified chamber (37°C, 30 min). Fluorescent images of five fields/section were obtained with Nikon A1 laser confocal microscope equipped with appropriate narrow band filter set with an excitation of 488 nm and an emission range of 574–595 nm. Fields were randomly chosen, avoiding the edges of the sections where autofluorescence was observed. Images were captured by the confocal laser microscope and were set by 20x objective lens at 1024 × 1024 pixels.

### 2.6. Determination of Indicators of Oxidative Stress

Following harvested brains, the tissues were immediately washed in chilled saline and then were homogenized in ice-cold saline for 20 min to prepare a 10% (w/v) homogenate. The homogenate was centrifuged at 2,500 rpm and 4°C for 15 min. The level of malondialdehyde (MDA), as well as the activities of superoxide dismutase (SOD), in the supernatant was investigated using a microplate reader (1510; Thermo Fisher Scientific, Waltham, MA, USA) according to the instructions provided with the assay kits (Nanjing Jiancheng Bioengineering Institute, Nanjing, China). The assay results were normalized to the protein concentration in each sample and expressed as U/mg protein or nmol/mg protein.

### 2.7. Western Blot

The prepared brain tissues were weighed and homogenized in 1 : 10 (w/v) ice-cold whole-cell lysis buffer (Nanjing KeyGen Biotech Co., Ltd., Nanjing, China) using a glass homogenizer. Soluble proteins were collected and centrifuged at 12,000 ×g for 10 min at 4°C. Tissue total protein concentrations were determined by a BCA Protein assay reagent kit (Beijing TransGen Biotech Co., Ltd.). Tissue total protein (50 *μ*L; p-ERK1/2, ERK1/2, caspase-3, Bax, Bcl-2, and GAPDH) was separated by 10% or 12% sodium dodecyl sulfate-polyacrylamide gel electrophoresis (SDS-PAGE) and then transferred onto a nitrocellulose membrane. The membrane was blocked with PBST containing 5% nonfat dry milk for 1 h and then incubated overnight at 4°C with the corresponding primary antibodies. The primary antibodies and dilutions were as follows: primary antibodies against phosphorylated ERK1/2 (number 9101, 1 : 1000), ERK 1/2 (number 4695, 1 : 1000), and GAPDH (number 5174, 1 : 1000) were purchased from Cell Signaling Technology (Danvers, MA), and caspase-3 (1 *μ*g/mL, number ab32351, 1 : 1000), Bax (number ab7977; 1 : 1000), and Bcl-2 (number ab7973; 1 : 100) were purchased from Abcam Plc, Cambridge, UK. After washing 3 times with PBST, the membrane was incubated with secondary antibody (goat anti-rabbit IgG, 1 : 10,000; Licor). The membrane was quantified using a western blotting detection system with a Li-cor Odyssey Scanner imaging densitometer, and the results of detected bands were quantified with Multi-Analyst software (Bio-Rad Laboratories).

### 2.8. Cell Apoptosis Assay

For TUNEL staining, paraffin blocks were cut into 5 *μ*m thickness coronal sections. To detect apoptotic cells, TUNEL staining was performed using an In Situ Cell Death Detection Kit (POD, ROCHE, cat.: 11684817910) according to the manufacturer's protocol. Briefly, the sections were deparaffinized in xylol, rehydrated by successive series of alcohol, washed in phosphate-buffered saline (PBS), and deproteinized (or permeabilized) by proteinase K (20 *μ*g/mL) for 30 min at room temperature. Then, the sections were rinsed and incubated with 3% H_2_O_2_ for 10 min in the dark to block endogenous peroxidase and rinsed with PBS; then, the sections were incubated in the TUNEL reaction mixture for 60 min at 37°C in light-protected and humidified atmosphere and rinsed with PBS. After that, the sections were rinsed with PBS and 50 *μ*L DAPI (4′,6-diamidino-2-phenylindole) was incubated for 5 min and rinsed with PBS and then all slides were mounted by cover slip and analyzed by fluorescence microscope. TUNEL labeling for cells death was normalized to DAPI staining for all cells. TUNEL controls were performed by incubating slides with 100 *μ*L label solution.

### 2.9. Double Fluorescent Staining

To clarify the spatial relationship between phospho-ERK1/2 expression and DNA fragmentation, we performed double staining for phospho-ERK1/2 and terminal deoxynucleotidyl transferase-mediated uridine 5′-triphosphate-biotin nick end labeling (TUNEL) using a fluorescent method. After deparaffinization and hydration, the sections in 10-mmol/L sodium citrate (pH 6.0) were boiled for 5 minutes at 95°C. Endogenous peroxidase was blocked with 0.3% hydrogen peroxidase for 10 min in the dark. The sections were incubated with PBS containing 10% normal goat serum at room temperature to eliminate any nonspecific binding and were incubated overnight at 4°C with monoclonal antibodies against p-ERK1/2 (1 : 100). Then, the sections were incubated with goat anti-rabbit IgG H&L (Alexa Fluor 647, ab150079) for 60 min. After that, TUNEL reaction mix was applied and incubated for 1 h. Then, the sections were rinsed with PBS and all slides were mounted by cover slip and analyzed by fluorescence microscope. Some sections were single labeled with TUNEL and DAPI for quantification of TUNEL positive cells.

### 2.10. Statistical Analysis

Values are expressed as mean ± standard error of the mean. All data were analyzed using one-way analysis of variance (ANOVA), followed by the least significant difference post hoc test (two-tailed). All statistical analyses were performed using SPSS software (version 6.0). *P* < 0.05 was considered to be statistically significant.

## 3. Results 

### 3.1. Baseline Characteristics

As Table 1 showed, there were no significantly different baseline parameters before CA induction among all groups, including body weights (BW), heart rate (HR), systolic pressure (SP), diastolic pressure (DP), and mean arterial pressure (MAP), the duration of transesophageal stimulation prior to CA (stimulation duration), and the duration of CPR prior to ROSC (CPR duration) (*P* > 0.05).

### 3.2. PD Improved Survival Rates and Neurologic Deficit Scores

Survival rates and neurologic deficit scores (NDS) of rats after ROSC were shown in Table 2. All sham rats lived to the observing end point of 72 h. Better survival rates of 12, 24, 48, and 72 h were seen in the PD groups compared with the CA group after ROSC (*P* < 0.05). Rats in the CA and PD groups exhibited a significant neurologic deficit and improvement over time, as compared with the sham group (no neuronal deficit; *P* < 0.05). Deficits were consistently less severe in PD group compared with CA group (*P* < 0.05 at 12, 24, and 48 h).

### 3.3. PD Decreased Brain ROS

To evaluate the effects of PD on ROS production induced by CA/CPR, the levels of ROS were measured by DHE staining after 12, 24, 48, and 72 h of reperfusion (Table 3). Compared to the sham-operated group, ROS were markedly upregulated (*P* < 0.05) in the CA group at 12, 24, 48, and 72 h of ROSC. Treatment of the rat with PD exerted antioxidant effects as evidenced by a decrease in the ROS levels (*P* < 0.05) after 12, 48, and 72 h of reperfusion while at 24 h of reperfusion treatment with PD showed no effects. Table 3 also indicated that the level of ROS increased at 12 h, reached peak levels at 24 h, and gradually decreased from 48 to 72 h of reperfusion in both CA and PD groups.

### 3.4. Antioxidant Activity of PD in Rat with Cerebral IR Injury

To evaluate the effects of PD on oxidative stress induced by CA/CPR, the levels of MDA and SOD activity were measured after 12, 24, 48, and 72 h of reperfusion (Table 4). Compared with the sham-operated group, SOD activity decreased significantly (*P* < 0.01) and the MDA level markedly increased (*P* < 0.01) in the CA group at 12, 24, 48, and 72 h of ROSC. However, treatment with PD restored an increase of SOD activity (*P* < 0.01) and a decrease in the MDA concentration (*P* < 0.01) after 12, 48, and 72 h of reperfusion while at 24 h of reperfusion treatment with PD showed no effects.

### 3.5. PD Decreased ERK1/2 Phosphorylation

**Figure 1:**
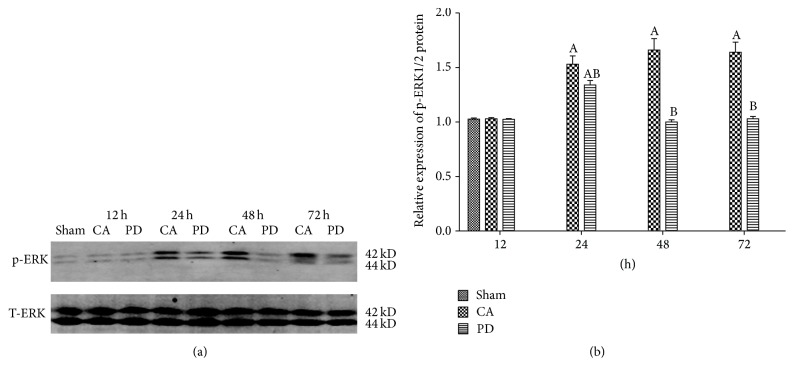
Time-course western blot analysis of p-ERK1/2 in cerebral ischemia reperfusion at 12 h, 24 h, 48 h, and 72 h. (a) Representative western blot. (b) Phosphorylation of ERK1/2 ratios (phosphorylated versus total protein). The phosphorylation of ERK1/2 increased significantly in all rats within 24 h after CA/PCR compared to sham group (*P* < 0.05). Compared to CA group, treatment with PD98059 significantly reduced phosphorylation of ERK1/2 from 48 h to 72 h of PCR, *P* < 0.05; mean ± standard deviation. *n* = 6 for each group (A = *P* < 0.05 versus the sham group; B = *P* < 0.05 versus the CA group).

We used western blot for analysis of phospho-ERK 1/2 and total ERK 1/2 expression. As shown in Figure 1(a), the bands of phospho-ERK1/2 and ERK1/2 were observed at 42 and 44 kDa in the whole-cell fraction from rat brains. There were no significant differences in p-ERK level between sham group and CA and PD groups at 12 h after ROSC. The phosphorylation of ERK1/2 increased significantly in rats subjected to CA/CPR at 24 h of reperfusion as compared to sham group (*P* < 0.05). The elevating of p-ERK 1/2 was sustained to 72 h in CA group. However, treatment with PD showed markedly decreased phosphorylation of ERK1/2 from 48 h to 72 h of reperfusion as compared to CA group (*P* < 0.05) (Figure 1(b)).

### 3.6. PD Decreased TUNEL Positive Neurons in Cortex

**Figure 2:**
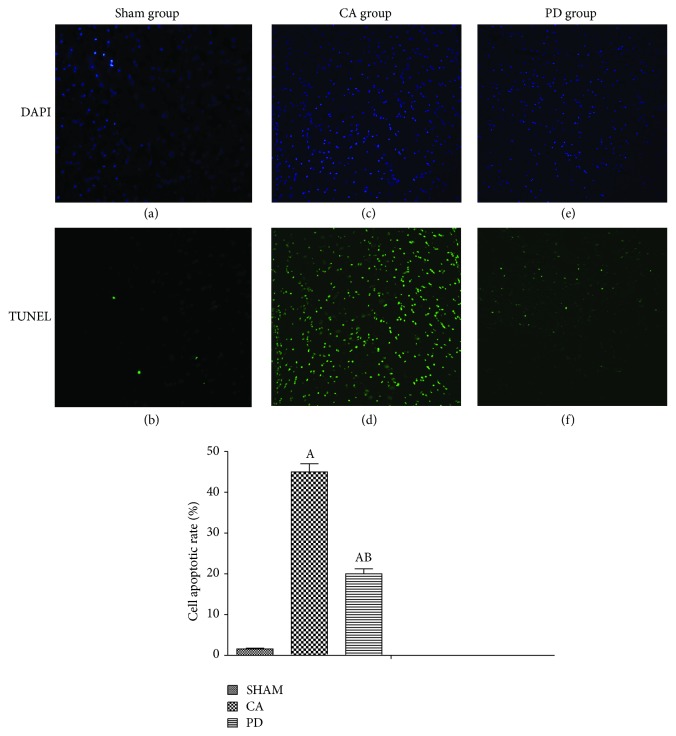
PD98059 decreased neuronal apoptosis in cortical brain. Apoptotic cells were induced after 72 h of CA/CPR. Quantitative analysis of TdT-mediated dUTP-biotin nick end labeling (TUNEL; green) positive cells or 4′,6-diamidino-2-phenylindole (DAPI; blue) to label all cells. Cells death increased significantly in the CA group and PD group as compared with sham group (*P* < 0.01). Treatment with PD98059 (PD group) induced a decreased apoptotic index (*P* < 0.01); mean ± standard deviation; *n* = 6 for each group.

Figure 2 presented the fluorescent signal of TUNEL and DAPI staining in cortex: TUNEL staining to monitor DNA damage, and DAPI staining to monitor morphological changes of nuclei. Cell apoptotic rate was defined as the percentage of TUNEL staining cells to DAPI staining cells. As Figures 2(a) and 2(b) showed, in the sham-operated group, there was a small amount of apoptotic cells in the cortex. Compared to the sham-operated group, the neuronal apoptotic rate was significantly increased at 72 h in rat brain subjected to CA/CPR (*P* < 0.01; Figures 2(c)-2(d) and 2(e)-2(f)). However, the increase in neuronal apoptosis was markedly reduced in the groups treated with PD (*P* < 0.01). These results suggested that the treatment with PD effectively prevented expansion of apoptotic cell death in CA/CPR model.

### 3.7. Double Labeling with Phospho-ERK Expression and DNA Fragmentation Detected by TUNEL Staining after CA/CPR

**Figure 3:**
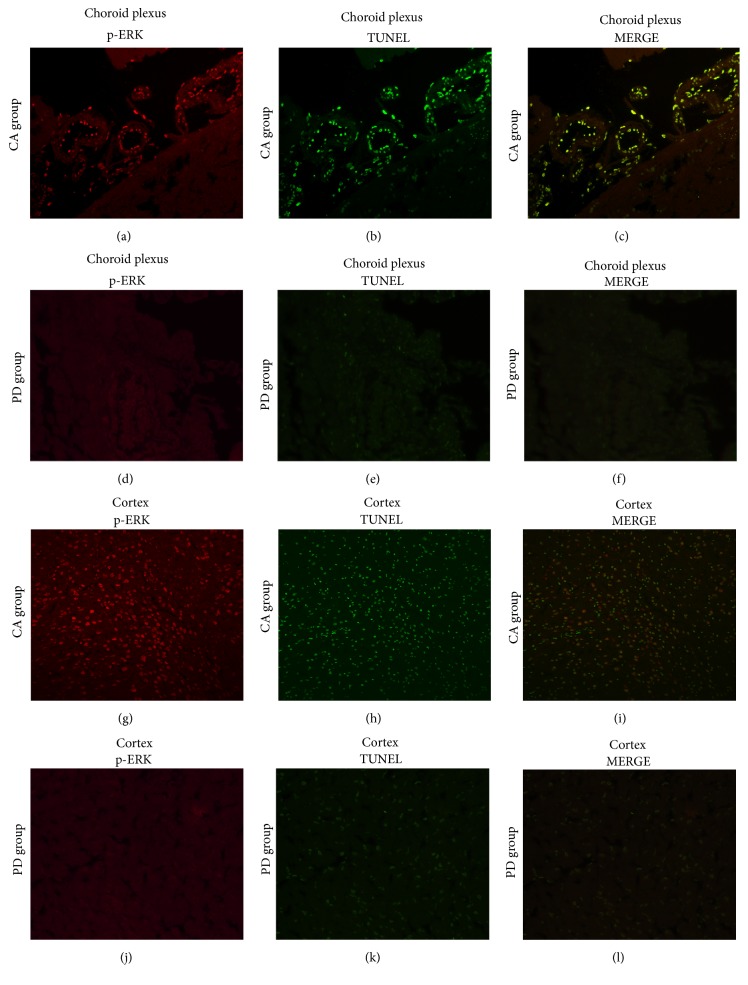
Representative photomicrographs show immunofluorescent staining for phospho-ERK and TUNEL. Phosphorylation of ERK1/2 (red) and TUNEL (green) in rat at 72 h induced CA/CPR. Sections were prepared from choroid plexus and cortex, magnification ×200; mean ± standard deviation; *n* = 6.

To corroborate p-ERK1/2 activation involved in cell death pathways, we examined the coexpression p-ERK1/2/TUNEL by double immunostaining with p-ERK1/2 and TUNEL.  Figure 3 demonstrated that the coexpression of the p-ERK1/2 and TUNEL staining is mainly distributed in choroid plexus (Figures 3(a), 3(b), and 3(c)) and in cortex (Figures 3(g), 3(h), and 3(i)) in CA rats at 72 h after ROSC. The average p-ERK1/2/TUNEL copositive cell rate was 57 ± 7.9% of TUNEL cells. There was no fluorescent signal of p-ERK1/2/TUNEL copositive cell be detected in PD-treated rats in choroid plexus (Figures 3(d), 3(e), and 3(f)) as well as in cortex (Figures 3(j), 3(k), and 3(l)). These results suggested that the phosphorylation of ERK1/2 may be associated with neuronal death pathway in CA/CPR rat model.

### 3.8. PD Decreased Cleaved Caspase-3

**Figure 4:**
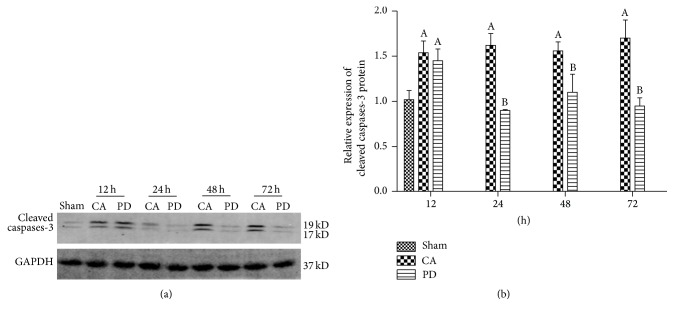
Effects of PD98059 (PD) on the expression of caspase-3. (a) Representative western blot of cleaved caspase-3 in brain of rat subjected to CA/CPR at 12, 24, 48, and 72 h following reperfusion. (b) Cleaved caspase-3 band intensity normalized to glyceraldehyde 3-phosphate dehydrogenase (GAPDH). Representative western blot showing the cleaved caspase-3 significantly increased in CA group and PD at 12 h after CA/PCR compared to sham operation (*P* < 0.05). The increase of cleaved caspase-3 was sustained to 72 h of reperfusion in CA. In contrast, treatment with PD98059 decreased significantly caspase-3 from 24 h to 72 h; mean ± standard deviation. *n* = 6 for each group (A = *P* < 0.05 versus the sham group; B = *P* < 0.05 versus the CA group).

The bands of cleaved caspase-3 were observed at 17 and 19 kDa in the whole-cell fraction from rat brains (Figure 4(a)). There was significant elevating of cleaved caspase-3 level between CA and PD group and sham-operation groups at 12 h after ROSC (*P* < 0.05). The increase of cleaved caspase-3 was sustained to 72 h in CA group. However, treatment with PD decreased significantly the cleaved caspase-3 from 24 h to 72 h of reperfusion as compared to CA group (*P* < 0.05) (Figure 4(b)).

### 3.9. PD Affects the Expression of Bax and Bcl-2 Protein

**Figure 5:**
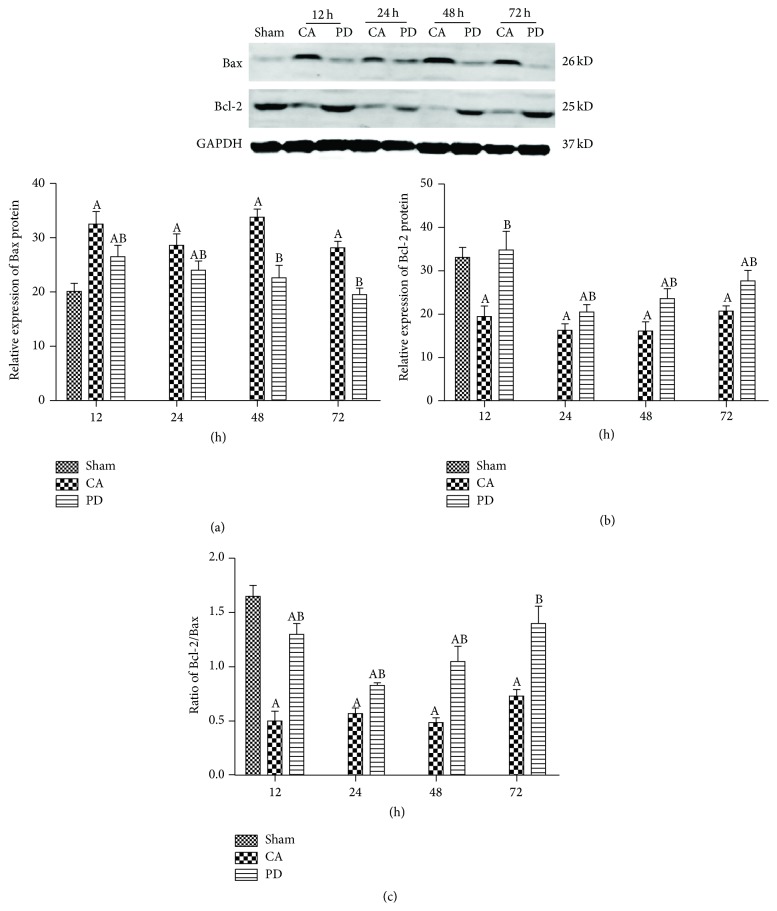
Effects of PD98059 (PD) on the expression of Bax and Bcl-2. (a) Representative western blot of Bax and Bcl-2 in brain of rat subjected to CA/CPR at 12, 24, 48, and 72 h following reperfusion. (b) Bax and Bcl-2 bands intensity normalized to glyceraldehyde 3-phosphate dehydrogenase (GAPDH); mean ± standard deviation; *n* = 6 for each group (A = *P* < 0.05 versus the sham group; B = *P* < 0.05 versus the CA group).

Compared with the sham-operated group, the CA group displayed a higher Bax protein level and a lower Bcl-2 protein at 12 h, 24 h, 48 h, and 72 h of ROSC (Figure 5, *P* < 0.05). However, treatment with PD resulted in a significant upregulation in Bcl-2 expression (*P* < 0.05) and a marked downregulation in Bax expression (*P* < 0.05) in rat subjected to CA/CPR. The Bcl-2/Bax ratio was significantly decreased (*P* < 0.05; Figure 5(c)) in CA group compared to the sham-operated group. However, the Bcl-2/Bax ratio increased from 12 to 48 h and returned to approximately normal levels at 72 h of reperfusion in the group treated with PD (*P* < 0.05; Figure 5(c)).

## 4. Discussion 

In the present study, we revealed a neuroprotective effect of PD against cerebral global ischemia. Treatment with PD downregulated the percentage of apoptotic cells and improved survival rate and neurological deficits by decreasing of ROS and ERK as well as apoptotic protein. The mechanism underline PD neuroprotection may be involved in the suppression of the oxidative stress production through the regulation of the expression in ROS, MDA, and SOD activity as well as the suppression of p-ERK and apoptotic biomarkers as evidenced by decreased caspase-3 and Bax and increased Bcl-2 in brain of rat subjected to CA/CPR.

CA rat models that closely mimic patient cardiac arrest circumstances are considered reliable and less invasive to study neurological deficit and the development of pathology in the brain after CA [19, 20]. The survival rates, percentage of apoptotic cells, and NDS are useful to estimate the potency of cerebral drugs in the treatment of postresuscitation brain injury from CA. In the present study, treatment with PD significantly downregulated the percentage of apoptotic cells and improved survival rates and NDS following CA/CPR in a rat model.

Many evidences indicated that excessive ROS production and subsequent oxidative stress play harmful roles during cerebral I/R injury [6, 7]. Overproduction of ROS can inflict direct damage to cellular molecules such as lipid, proteins, and nucleic acids in the ischemic tissue, leading to membrane injury and cell death [8, 24]. Lipid peroxidation is known to be one of the primary pathophysiological mechanisms, which is also implicated in cerebral I/R injury [25]. Under physiological conditions, there is a low concentration of lipid peroxidation in brain tissue. By contrast, under oxidase stress, the cells may produce a high concentration of lipid peroxidation and ROS because the cells induce apoptosis or necrosis programmed cell death [26]. Moreover, the brain tissue is composed of high level of polyunsaturated fatty acids in membrane [27], which makes it very sensitive to lipid peroxidation [26]. As a final product of lipid peroxidation, MDA is one of the most preferred markers for oxidative stress which results in cytotoxic effects and neuronal death [28]. By contrast, SOD is an enzyme present in all oxygen-metabolizing cells, which plays an important role in the maintenance of low concentrations of oxidants and redox homeostasis in tissue through the scavenging of oxidants, preventing harmful ROS generation [29]. Our results indicated that, from 12 h to 72 h of ROSC, brain tissue markedly increased ROS and MDA and decreased SOD activity levels in rat subjected to CA/CPR. However, treatment with PD exerted antioxidant effects as evidenced by downregulation of ROS and MDA and restored upregulation of SOD expression.

Oxidative stress induced cell apoptosis through activation of some cellular signal pathways; for example, H_2_O_2_ can stimulate the Ras/Raf/ERK pathway by increasing the activation of tyrosine kinase receptors, such as platelet-defined growth factor receptor or EGF receptor [30, 31]. Consistent with this finding, in present study indicated that at 24 h of reperfusion, when the ROS at highest level may be associated with initial increase of ERK phosphorylation. The activation of ERK 1/2 signaling pathway also plays a significant role in endothelial cell injury after oxygen-glucose deprivation through vascular endothelial growth factor (VEGF) [32]. Persistent activation of ERK1/2 induced increased glutamate oxidative toxicity and H_2_O_2_ generation leads to cortical neuronal cells death [33, 34]. Our results indicate that prolonged activation of ERK from 24 h to 72 h may be involved in microvessel and cortical neuron cell death in rat subjected to CA/CPR (Figure 3). Moreover, coexpression of p-ERK/TUNEL showed that cell death of the microvessels mainly distributes in the choroid plexus of lateral ventricle. The choroid plexus is a free-floating organ located in the roof of the lateral ventricles and constitutes an essential part of the blood brain barrier (BBB) [35]. The BBB plays important roles in the maintenance of central nervous system homeostasis; its disruption contributes to oxidative stress and neuronal damage [36]. The inhibition of ERK pathway with PD98096 or U0126 decreases ROS production by inhibition of glutamate toxicity, suggesting this effect is involved in protection of neurovascular system and neuron in cerebral I/R [32, 33]. In fact, the data of our study also indicate that treatment with ERK inhibition exerts neuroprotective effects by suppression of the increase of ROS/p-ERK as well as cells death.

ROS generation during cerebral ischemia reperfusion acts as upstream signaling molecules that initiate cell death [37, 38]. The increase of ROS generation is involved in increased intracellular Ca(2+) concentration and alterations of mitochondrial membrane potential [38]. The subsequent translocation of Bax to the mitochondria results in release of cytochrome c and activation of caspases [39]. Caspases are well-known drivers of apoptotic cell death, cleaving cellular proteins that provide critical links in cell regulatory networks controlling dying cell [40]. Active caspase-3 leads to DNA fragmentation, formation of apoptotic bodies, and neuronal cells death [41]. By contrast, as an antiapoptotic protein, the Bcl-2 expression is maintained at relatively high levels in neuron [42] and helps preserve the mitochondrial integrity by suppressing the release of cytochrome c [43]. Bcl-2 overexpression in neurons reduces caspase-3 activation and rescues cerebellar degeneration [44]. Moreover, high level of ROS can regulate cell death by decreasing expression of Bcl-2 [45]. Thus, inhibition of Bcl-2 expression was known to induce death of a variety of neuronal cell lines [42]. In this study, we found that brain tissue significantly increased ROS and Bax and decreased Bcl-2 and Bcl-2/Bax ratio in rat subjected to CA/CPR. The results which may explain the increased number of apoptotic neurons in rat induced CA/CPR. Treatment with PD reduced these effects. Taken together, we suggest that PD downregulated Bax and caspase-3 and upregulated Bcl-2 may be associated with reducing ROS generation.

## 5. Conclusions 

In conclusion, the findings of present study show that ROS and ERK 1/2 signaling pathway play a significant role in cerebral injury after CA/CPR. ERK inhibitor can be considered to explore for the treatment of brain injury resulting from cardiac arrest.

## Figures and Tables

**Table 1:** Baseline parameters.

Group	*n*	BW (g)	HR (beats/min)	SP (mmHg)	DP (mmHg)	MAP (mmHg)	Stimulation duration (s)	CPR duration (s)
Sham	6	355.76 ± 46.21	424.60 ± 33.96	112.37 ± 10.47	91.31 ± 7.51	96.24 ± 7.39	—	—
CA	26	386.36 ± 44.53	412.48 ± 37.14	110.57 ± 9.17	90.74 ± 8.84	98.51 ± 8.87	80.16 ± 9.07	118.18 ± 50.30
PD	26	363.60 ± 47.14	418.58 ± 39.46	111.89 ± 10.66	91.88 ± 9.96	97.53 ± 9.66	82.57 ± 10.20	117.44 ± 48.77

**Table 2:** The survival rate and neurologic deficit scores (NDS) after ROSC.

Group	12 h		24 h		48 h		72 h	
	Survival rate	NDS	Survival rate	NDS	Survival rate	NDS	Survival rate	NDS
Sham	6/6 (100%)	80.00 ± 0 (*n* = 6)	6/6 (100%)	80.00 ± 0 (*n* = 6)	6/6 (100%)	80.00 ± 0 (*n* = 6)	6/6 (100%)	80.00 ± 0 (*n* = 6)
CA	10/13 (76.9%)	47.94 ± 5.83^*∗*^ (*n* = 10)	6/13 (46.2%)^*∗*^	53.26 ± 2.96^*∗*^ (*n* = 6)	4/13 (30.8%)^*∗*^	58.62 ± 3.33^*∗*^ (*n* = 4)	3/13 (23.1%)^*∗*^	73.55 ± 2.15 (*n* = 3)
PD	13/13 (100%)^#^	62.77 ± 357^*∗*#^ (*n* = 13)	10/13 (76.9%)^#^	67.71 ± 3.23^*∗*#^ (*n* = 10)	8/13 (61.5%)^*∗*#^	72.46 ± 2.44^*∗*#^ (*n* = 8)	5/13 (38.5%)^*∗*^	75.25 ± 1.68 (*n* = 5)

^*∗*^ *P* < 0.05 versus sham group; ^#^ *P* < 0.05 versus CA group (*n* = 6).

**Table 3:** The ROS level of rat brain after ROSC.

The average fluorescence intensity of rat brain after ROSC					
Group	*N*	12 h	24 h	48 h	72 h
Sham	6	387.85 ± 35.54	387.85 ± 35.54	387.85 ± 35.54	387.85 ± 35.54
CA	24	681.31 ± 21.11^*∗*^	722.27 ± 34.17^*∗*^	697.38 ± 28.58^*∗*^	655.33 ± 24.65^*∗*^
PD	24	371.70 ± 10.30^#^	390.80 ± 33.50^#^	386.31 ± 26.24^#^	376.67 ± 37.71^#^

^*∗*^ *P* < 0.05 compared to the sham group; ^#^ *P* < 0.05 compared to CA group (*n* = 6).

**Table 4:** The MDA and SOD activity levels in brain after ROSC.

Group	*N*	12 h		24 h		48 h		72 h	
		MDA levels (U/mg prot)	SOD activity (nmol/mg prot)	MDA levels (U/mg prot)	SOD activity (nmol/mg prot)	MDA levels (U/mg prot)	SOD activity (nmol/mg prot)	MDA levels (U/mg prot)	SOD activity (nmol/mg prot)
Sham	6	4.67 ± 0.66	120.67 ± 8.96	4.67 ± 0.66	120.67 ± 8.96	4.67 ± 0.66	120.67 ± 8.96	4.67 ± 0.66	120.67 ± 8.96
CA	24	7.47 ± 6.01^*∗∗*^	75.16 ± 4.85^*∗∗*^	8.38 ± 0.75^*∗∗*^	61.65 ± 8.56^*∗∗*^	10.19 ± 0.70^*∗∗*^	54.13 ± 5.81^*∗∗*^	7.50 ± 0.729^*∗∗*^	60.67 ± 7.17^*∗∗*^
PD	24	5.82 ± 0.80^*∗*##^	97.33 ± 7.02^*∗∗*##^	8.18 ± 0.84^*∗∗*^	70.60 ± 8.47^*∗∗*^	5.19 ± 0.62^##^	84.12 ± 7.21^*∗∗*##^	5.03 ± 0.71^##^	83.24 ± 7.03^*∗∗*##^

^*∗*^ *P* < 0.05 and ^*∗∗*^ *P* < 0.01 compared to the sham group; ^##^ *P* < 0.01 compared to CA group (*n* = 6).
